# HA117 gene increased the multidrug resistance of K562 cells in vitro: an investigation to the function of a novel gene related to drug resistance

**DOI:** 10.1186/1756-9966-28-63

**Published:** 2009-05-12

**Authors:** Yuxia Guo, Gaihuan Zheng, Xianqing Jin, Youhua Xu, Qing Luo, Xiaomei Liu, Zhenzhen Zhao, Yong Chen

**Affiliations:** 1 The Affiliated Children's Hospital, Chongqing Medical University, PR China

## Abstract

**Objective:**

A novel multi-drug resistance gene named as HA117 has been screened and cloned in multidrug resisitant leukemia cell lines in our previous research, but its function is still unknown. In this study, HA117 gene was investigated whether it could increase the drug resistance in chronic myelogenous myeloid leukemia cell line K562.

**Methods:**

HA117 was cloned and adenovirus vectors were constructed with the HA117 gene (Adeasy-HA117). K562 cells were infected by Ad-HA117 to get the K562/Ad-HA117 cells with HA117 gene expression. The infection efficiency and the multiplicity of infection (MOI) were detected by fluorescence and flow cytometry. The expression of HA117 gene was detected by RT-PCR. The drug sensitivities of K562/Ad-HA117 cells were detected by Methyl Thiazolyl Tetrazolium (MTT) assay.

**Results:**

Recombinant adenovirus vectors were constructed and a MOI of 100 is most suitable to infect K562 cells. The infected K562 cells demonstrated in vitro production of HA117 mRNA as measured by reverse-transcriptase polymerase chain reaction. There were no significant changes in K562/Ad-HA117 cells growth, while the drug sensitivities of K562/Ad-HA117 cells to Vincristine, Adriamycin, Etoposide, Daunorubicin, Mitomycin and Cyclophosphamide decreased 4.44, 7.18, 3.01, 9.53, 3.48 and 3.61 times than that of uninfected K562 cells, respectively (P < 0.05).

**Conclusion:**

Expression of the novel gene HA117 could significantly increased the multi-drug resistance of K562 cells, which indicated that HA117 is a functionally relevant multidrug resistance gene.

## Introduction

Multi-drug resistance (MDR) of tumor cells, including leukemia cells, is a defense mechanism for retaining homeostasis when they are damaged by cytotoxic drugs [1]. Tumor cells emerge a series of biological changes during the development of MDR in them. In molecular mechanism, occurrence of tumor cells' MDR is because of expression of genes related drug resistance [2]. To investigate which genes were in regulation in MDR of tumor cells, we established the multi-drug resistance cells HL-60/MDR using acute myelocytic leukemia cell line HL-60 at previous study. Then we screened and cloned the MDR related genes in HL-60/MDR cells using differential hybridization and gene chip [3,4] and found a novel gene HA117 (GeneBank: AY230154) which may be related to MDR[5]. In this study, adenovirus vectors were constructed with the HA117 gene (Adeasy-HA117) to investigate whether HA117 gene could increase the drug resistance in chronic myelogenous myeloid leukemia cell line K562.

## Methods

### Reagents

Restriction enzymes and T_4 _ligases were bought from New England Biolabs company, USA. DNA polymerase, TaKaRa MiniBEST Plasmid Purification Kits, Agarose Gel DNA Fragment Recovery Kits, RNAiso reagents, Reverse Transcription PCR kits and primers were products of TaKaRa Biotechnology (Dalian, China) CO., LTD. Lipofectamine was from Invitrogen company, USA. The High-quality fetal bovine serum and 1640 medium were products of Gibco Company, USA. Vincristine (VCR) was offered by HuaLian Limited Company, ShangHai, China. Adriamycin (ADM) was produced by AIBAO pharmaceutical factory, Italy. Mitomycin was bought from Sigma Company, USA. Etoposide was offerd by LianYunGang pharmaceutical factory, China. Cytoxan was bought from SuHeng pharmaceuticalfactory, JangSu, China. Daunorubicin (DNR) was from Pharmacia Company, Italy.

### Plasmids and cell lines

BJ5183 strain, shuttle plasmid pAdTraek-CMV with Green Fluorescent Protein (GFP), adenoviral genome plasmid pAdeasy-1 and 293 cells were given by professor Tong-Chuan He in the molecular Oncology Laboratory of Chicago University, USA. The plasmid PUC57-HA117 containing HA117 gene, E. coli DH5α, and K562 cells were stored in our laboratory.

### Construction of recombined adenovirus Ad5-HA117[6]

Adenoviral shuttle plasmid pAdTrack-CMV and PUC57-HA117 were incised by restriction enzyme HindIII and KpnI. After incised, HA117 gene and pAdTrack-CMV were recovered using Agarose Gel DNA Fragment Recovery Kit, then linked by T4 joinase and transduced into E. coli DH5α. The transformed positive clone pAdTrack-HA117 was selected and identified by incision enzyme and sequence analysis. The pAdTrack-HA117 DNA was made to be inlinearization by PmeI cutting and transformed into adenoviral homologous shuttle plasmid BJ-Adeasy in a CaCl_2 _precipitational way. Positive clones BJ-Adeasy-HA117 were selected and transformed into competent cell DH5α. Then Adeasy-HA117 was verified by Pac1 digesting and packaged to be complete recombined adenovirus Ad5-HA117 in 293 cells. The first generation 293 cells were harvested and freezing-dissolved with solid carbon dioxide three times when they were floating after transfected 10–14 days. Supernatant containing virus was collected and infected 293 cells to amplify the recombined adenovirus massively. After amplified three turns and purified with density gradient centrifugation, high titer recombined adenovirus Ad5-HA117 was harvested and stored in -80°C to be used.

### Ad5-HA117 infected K562 cells in vitro

Human leukemic cells K562 were cultured were cultured in 37°C in RPMI 1640 cell culture medium containning 10% fetal calf serum. The cells in logarithmic phase were divided into 3 groups. The cells infected by Ad-HA117 were designed as experimental group and labeled as K562/Ad-HA117. The cells infected by empty ecombined adenovirus were control group and labeled as K562/Ad-null. The cells uninfected were designed as blank control group and labeled as K562. After cultured for 16 hours, cells concentration was modulated to be 2.0 × 10^5^/L with 1640 medium conaining 10% fetal bovine serum. Experimental groups were set up according to different multiplicity of infection (MOI). MOIs of each groups were 1, 10, 50, 100, 500 and 1000. Every group set up 6 pores. The efficiency of infection was detected using fluorescence microscope at 24 hours after infection.

### Reverse transcriptase-polymerase chain reaction (RT-PCR) for HA117 gene in K562 cells

Total cellular RNA was isolated from k562/Ad-HA117 cells, K562/Ad-null cells and K562 cells using RNAiso reagents at 24 hours after infection, respectively. The RT-PCR reactions were carried out using Reverse Transcription PCR kit. The upstream primer of β-actin was 5'-CTTTGGTATCGTGGAAGGACTC-3', and the downstream primer was 5'-AGTGGGTGTCGCTGTTGAAGT-3'. The upstream primer of HA117 gene was 5'-CAGAGTCAGGGACTTCAGCCTTAT-3', and the he downstream primer was 5'-CTGTTTCCTTCTCACTCCCAACCA-3'. The PCR was performed with a fist denaturation step at 94°C 5 minutes and 35 cycles of denaturation at 94°C for 1 minute, annealing at 68°C for 30 seconds and at 72°C for one minute. The PCR reaction products were detected with gel electrophoresis and ultraviolet transillumination.

### MTT assays for drug sensitivity

The drug sensitivity of experimental cells to 5-fluorouracil was determined by MTT assay at 24 hours after infection. Cell suspension was collected into 96-well flat-bottomed microtitre plates (1 × 10^5 ^cells/well). 6 concentrations of 5-fluorouracil were chosen according to preliminary experiment and were added to wells of culture plate containing 200 *μ*l cell suspension. After cultured at 37°C for 24 hours, 50 *μ*l of MTT solution (5.0 mg ml^-1^) were added to each well and incubated for 4 hours. Then the mixture containing the medium, drug, and unconverted MTT was removed carefully. DMSO was added to each well to dissolve the formazan and absorbance was read at 450 nm using a spectrophotometric microplate reader (SunRise, Austria). The survival rate of tumor cells for each concentrations was calculated following the formula: survival rate (%) = (1- ODdrug/ODcontrol) × 100. The 50% inhibiting concentration (IC_50_) of chemotherapeutic drugs was calculated according to the suvival rate for each concentration. The drug-resistant factor (RF), also named drug-resistant index, was calculated with the following formula: RF = experimental cells'IC_50_/control cells'IC_50 _[7]. All experiments were performed in triplicate.

### Drug Elimination Experiments

Cells (2.0 × 10^6^/L) in each group were incubated with Daunomycin (7.5 μg/L) for 30 min and observed under a fluorescence microscope. Then, cells were centrifugated and the supernate were used to determine the concentrations of daunomycin by flow cytometry.

### Statistical Analysis

The results were given as mean ± standard deviation. Differences in means of normally distributed data were assessed by Student's t test with Bonferroni correction. *P *value less than 0.05 is considered significant.

## Results

### Results of recombined adenovirus Ad5-HA117 construction

**Figure 1:**
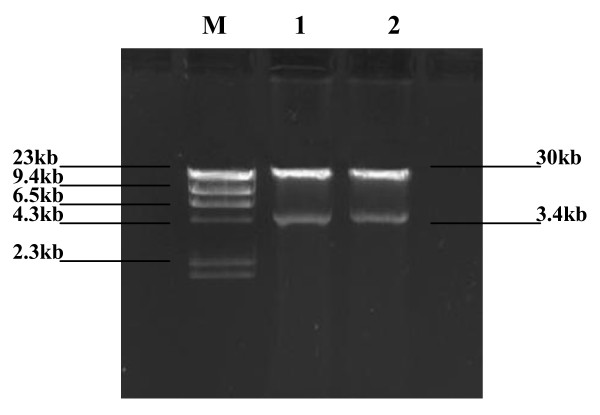
**Gel screening of Adeasy-HA117 after digested by Pac I**. After digeted with Pac I, Adeasy-HA117 produced 4.5 kb DNA strap, which proved that the homologous recombination was successful. M: DNA Marker; **1,2: **Adeasy-HA117

**Figure 2:**
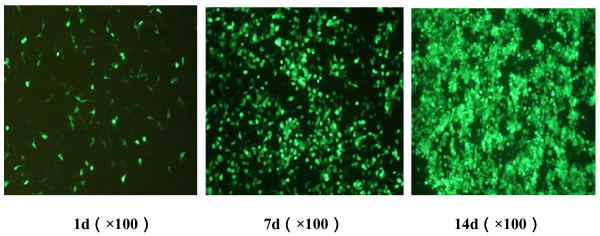
**The generation of recombinated adenovirus pAdeasy-HA117**.

By digested with HindIII and KpnI, a piece of 1.1 kb nucleotides (HA117 gene) was obtained and sequenced, which indicated that the recombined plasimid pAdTrack/HA117 was constructed successfully. The pAdTrack-HA117 was homologous recombined with BJ-Adeasy in E. coli. Then, the recombined Adeasy-HA117 plasmid was identified by Pac1 cutting. One 30 kb strap and one 4.5 kb strap could be seen by agarose gel electrophoresis, which proved that the homologous recombination was successful (Figure 1). Then, pAdeasy-HA117 was transfected into 293 cells. After two weeks, the transfected 293 cells became to be float from adherence observed by the GFP fluorescence intensity (Figure 2). At this time, the completed recombined adenovirus Ad5-HA117 was harvested.

### Expression of fluorescence and most suitable adenovirus amount of infetected K562 cells

**Figure 3:**
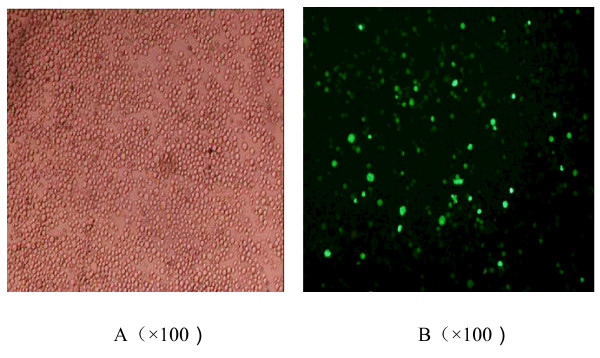
**Fluorescent expression of K562 cells after transfected 24 hours**. A:K562 cells; B: K562/Ad-HA117 cells expressed green fluorescence.

**Figure 4:**
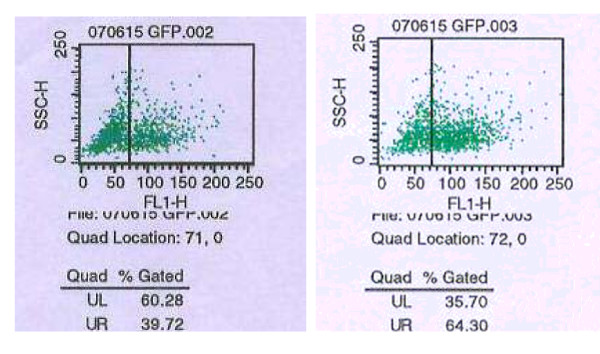
**The infection rates of K562 cells during different MOI detected by FCM**. The infection rates were about 39.72%~64.3%. A: MOI = 100; B: MOI = 1000.

The K562 cells had green fluorescent expression at 24 hours after infected (Figure 3). It was found that the infection rate of adenovirus to K562 cells increased with the adenovirus amout increased. Both cells' survival rate (exceeded 80%) and infection rate (reached 39.72%) were fairly well when MOI was 100. And the weak and dead cells increased obviously when MOI exceeded 100. So MOI 100 was chosen as the most suitable amount for the further investigation (Table 1 and Figure 4).

**Table 1:** The rates of infection and survival of cell during different MOI

	**MOI**					
	
	1	10	50	100	500	1000
**Infection rates**	**0.47 ± 0.04**	**5.83 ± 0.07**	**10.65 ± 0.11**	**16.19 ± 0.31**	**20.27 ± 0.52**	**30.42 ± 2.31**
**Survivil rates**	**90.33 ± 1.21**	**85.27 ± 1.37**	**82.11 ± 1.63**	**81 ± 1.42**	**62.23 ± 2.15**	**40.25 ± 2.13**

### RT-PCR results for HA117 gene expression in k562 cells

**Figure 5:**
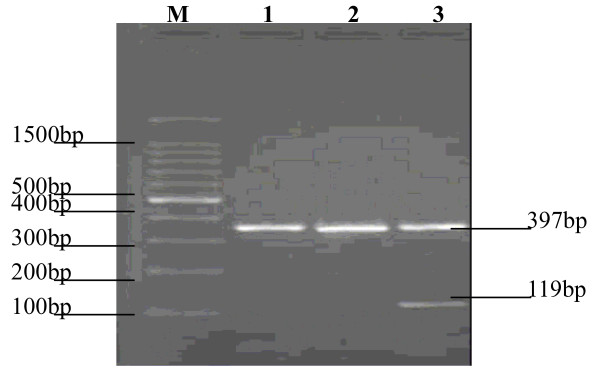
**The expression of HA117 gene mRNA in K562 cells**. M: DNA marker; 1:K562 cells; 2: K562/Ad-null cells had no HA117 gene expression; 3:K562/Ad-HA117 cells had HA117 gene expression. The DNA strap having 397 bp was β-actin.

Both uninfected K562 cells and K562/Ad-null cells had no HA117 gene expression, and HA117 expressed only in the K562/Ad-HA117 cells, which indicated that K562 cells could express exogenous HA117 gene when infected by Ad-HA117 (figure 5).

### The MTT assays results for K562 cells' drug sensitivity

The survival rates of K562/HA117 cells increased than that of K562 cells and K562/Ad-null cells. The RFs of K562/Ad-HA117 cells to VCR, ADM, Vp-16, DNR, MMC and CTX were 4.44, 7.18, 3.01, 9.53, 3.48 and 3.61 times than that of uninfected K562 cells, respectively (P < 0.05). And there was no significantly difference in RFs between the uninfected K562 cells and K562/Ad-null cells. It demonstrated that exogenous HA117 gene could induce K562 cells to develop drug resistance to the chemotherapeutic drugs (Table 2).

**Table 2:** The drug senstivity experimental results of K562 cells

**Drugs**	**inhibitory concentration(IC50)**		
	
	K562	*K562/Ad-HA117	*K562/Ad-null
VCR	0.052 ± 0.009	0.810 ± 0.060	0.031 ± 0.010
ADM	0.203 ± 0.018	0.985 ± 0.12	0.210 ± 0.014
VP-16	3.221 ± 0.021	7.834 ± 0.002	3.132 ± 0.031
DNR	0.089 ± 0.025	0.654 ± 0.203	0.091 ± 0.013
MMC	3.421 ± 0.215	11.023 ± 0.542	3.203 ± 0.189
CTX	1.654 ± 0.104	5.003 ± 0.006	1.721 ± 0.056

notice: *P < 0.05, compared with K562 and K562/Ad-null.

### HA117 gene was no drug-excretion function

**Figure 6:**
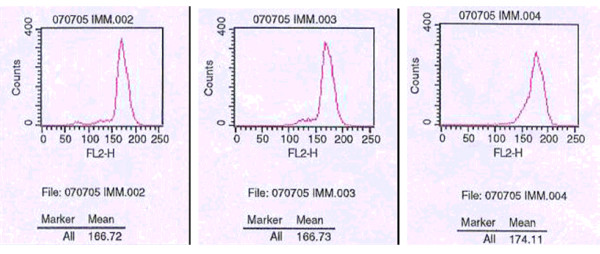
**Fluorescence intensity of DNR in K562 cells**. A: K562 cells, B: K562/Ad cells, C: K562/Ad-HA117 cells.

Daunorubicin was one kind of anti-cancer drugs which had autofluorescence. The drug's concentration in the cells could be determined directly by fluorescence intensity with a fluorescent microscope. There was no significant difference in the fluorescence intensity between the experimental group and control group, which indicated that HA117 gene had no drug-excretion function (Figure 6).

## Discussion

All-trans retinoic acid (ATRA) has been proposed as an alternative therapy for acute promyelocytic leukemia (APL), which is a specific subtype of acute myeloid leukemia (AML) (AML, M3) characterized by a chromosomal translocation t (15; 17) involving the promyelocytic leukemia (PML) gene on chromosome 15 and the retinoic acid receptor-alpha (RARa) gene on chromosome 17, Since 1988[8,9], we and others have shown that a high proportion of APL patients achieve complete remission (CR) with ATRA alone[10,11]. Unfortunately, further clinical experience has shown that they do not remain in long-term remissions if maintained on ATRA therapy alone [12]. When relapse occurs shortly after ATRA withdrawal in APL, the ATRA fails to induce a second remission and the APL cells develop drug resistance to other chemotherapeutics[13]. The exact mechanism is still unknown and there are some putative mechanisms for this phenomenon involving in overexpression of MN1 [14], mutations of RARa as noted in HL-60 cells[15], selection of non-APL leukemia clones and increased expression of proteins involved in ATRA's metabolism[16,17]. In our previous researches, we have established the suppressive subtractive hybridization library of the multi-drug resistance cell line HL-60/MDR inducing by ATRA to investigate the mechanism of MDR in APL cells. 12 MDR related genes with significant differential expression have been screened out to homology analysis. Of these, 11 matched known genes and the rest one showed no significant homology to human or non-human known sequences. It was named as gene clone HA117, but its function is unkown.

As we know, the recombined adenovirus vector is one of the vectors most commonly used for gene therapy. Compared to other viral vectors, it offer many advantages including relatively low pathogenicity in humans, wide host range and high replication efficiency[18,19]. Therefore, we selected the improved plasimid pAdeasy to construct the recombined adenovirus Ad-HA117 containing HA117 gene and K562 cells were infected by Ad-HA117 to get the K562/Ad-HA117 cells with HA117 gene expression. The infection efficiency and the multiplicity of infection (MOI) were detected by fluorescence and flow cytometry, it was found that the infection rate of adenovirus to K562 cells increased with the adenovirus amout increased and the weak and dead cells increased obviously when MOI exceeded 100. So MOI 100 was chosen as the most suitable amount for the further researches (Table 1 and Figure 4). We also found that HA117 expressed only in the K562/Ad-HA117 cells and exogenous HA117 gene could induce K562 cells to develop drug resistance to the chemotherapeutic drugs such as adriamycin, vinblastine, mitoxantrone and etoposide. But HA117 gene had no drug-excretion function

In conclusion, we constructed the recombined adenovirus Ad-HA117 which could express the novel gene HA117 and its expression could significantly increased the multi-drug resistance of K562 cells. It indicated that HA117 is a functionally relevant multidrug resistance gene. But whether HA117 could increase the drug resistance of tumor cell in vivo needs further study.

## Competing interests

The authors declare that they have no competing interests.

## Authors' contributions

YG constructed the recombined adenovirus and the MTT experiments and carried out the acquisition, analysis and interpretation of datas. GZ drafted and revised it critically for important intellectual content the article. YX directed the conception and designed of the study and final approval of the version to be submitted. XJ conceived of the study, and also designed of the study and final approval of the version to be submitted. QL directed and helped to the gene clone experiment. XL assisted to acquisition, analysis and interpretation of datas. ZZ assisted to construction of the recombined adenovirus and the MTT experimentsYC assited to drafted and revised the article. All authors read and approved the final manuscript.
